# Effect of moderate hyperbilirubinemia on an infant’s brain: a quantitative susceptibility mapping and ^1^H-MRS study

**DOI:** 10.3389/fped.2025.1464850

**Published:** 2025-03-27

**Authors:** Ru Zhao, Jia-Jia Xu, Lian-Zi Su, Yan-Qi Shan, Hao Zhan, Qun Pei, Long-Sheng Wang, Li-Wei Zou

**Affiliations:** ^1^ Department of Radiology, The Second Affiliated Hospital of Anhui Medical University, Hefei, China; ^2^ Department of Pediatrics, The Second Affiliated Hospital of Anhui Medical University, Hefei, China; ^3^ MOE Key Laboratory of Population Health Across Life Cycle and Anhui Provincial Key Laboratory of Population Health and Aristogenics, Hefei, China

**Keywords:** NHB, QSM, MRS, PLSC, TSB

## Abstract

**Objective:**

The effects of moderate neonatal hyperbilirubinemia (NHB) remain unknown. The aim of this work was to investigate whether moderate NHB has an impact on an infant’s brain and explore the relationship between brain magnetic susceptibility, brain metabolites, and biochemical tests in moderate NHB using quantitative susceptibility mapping (QSM) and magnetic resonance spectroscopy (MRS).

**Materials and methods:**

In total, 28 term babies with moderate levels of blood bilirubin were enrolled in the NHB group, and 16 term infants were enrolled in the control group. All the patients underwent biochemical tests, 1H-MRS, and QSM examinations. Biochemical test results [e.g., direct bilirubin (DBiL)], metabolite ratios [e.g., glycerophosphocholine (GPC)], and susceptibility values were collected. The Mann–Whitney *U*-test was used to assess the differences between the NHB and control groups. Partial least square correlation (PLSC) analyses were performed to analyze the correlations between the biochemical results and the metabolite ratios and susceptibility values.

**Results:**

The Mann–Whitney *U*-test showed that significant differences were observed in the biochemical results, susceptibility values of the left putamen, and absolute concentrations of GPC between the NHB group and the controls. No significant differences were found in the metabolite ratios between the two groups. The PLSC analysis demonstrated that the ratios of myo-inositol (Ins), N-acetylaspartate (NAA), and GPC relative to creatine and phosphocreatine had a robust correlation with DBiL in the NHB group. Furthermore, increasing susceptibility values of putamen, globus pallidus, caudate nucleus, and thalamus had a moderate correlation with decreasing DBiL and increasing TSH concentrations in the NHB group.

**Conclusion:**

This study demonstrated that moderate hyperbilirubinemia could induce metabolic and susceptibility changes in an infant’s brain (e.g., decreased susceptibility values and metabolite values) and these changes have a correlation with biochemical test results.

## 1 Introduction

Neonatal hyperbilirubinemia (NHB) is a remarkable increase in the circulation of total serum bilirubin (TSB) and is usually accompanied by jaundice. NHB is the leading cause of emergency department visits and hospitalization during the neonatal period (1). High TSB concentrations may result in bilirubin encephalopathy (NBE) and kernicterus, which can lead to neurological damage (2), including hearing loss, impairment of upwards gaze, and athetoid cerebral palsy. Most physicians have adopted the use of TSB levels >20 mg/dL as an indication of vulnerability to neurotoxicity (3, 4). However, it is free bilirubin that, unconjugated to albumin, can enter the brain and induce brain toxicity. Currently, there is no method for the routine quantification of free bilirubin (5). It has been proven that severe hyperbilirubinemia can lead to chronic bilirubin encephalopathy and kernicterus while the effects of moderately high bilirubin levels are still unknown. Though a risk threshold for TSB has been proposed (4, 6), the minimum amount of bilirubin that can cause damage or whether moderate hyperbilirubinemia has an impact on brain function still needs to be elucidated. Previous studies (7, 8) suggest that moderate hyperbilirubinemia may have a correlation with minor behavioral effects and delayed motor development. Thus, to avoid these delayed deficits, the early detection of abnormal moderate NBE-induced brain dysfunction and early intervention may be needed.

Magnetic resonance imaging (MRI) plays an important role in the diagnosis of NBE and kernicterus. Increased signal intensity in the bilateral globus pallidus and sometimes in the subthalamic nucleus on T1-weighted images (T1WI) has been reported as an imaging feature of NBE. However, T1WI hyperintensity is a transient phenomenon in NBE and most neonates with NBE present with normal MRI T1WI signals (9). Thus, high TSB-induced neurotoxicity sometimes is invisible in ordinary MRI scans. ^1^H MR spectroscopy (MRS) is valuable as it depicts metabolic changes in the brain (10, 11). Some of the neurotoxic effects (12) of bilirubin are gliosis, demyelination, and inhibition of glutamate uptake by astrocytes in the basal ganglia area, which could be depicted by ^1^H-MRS. Studies (11, 13, 14) have shown that the brain metabolism changes in patients with NBE or kernicterus through comparison with normal groups. However, few studies have discussed the ^1^H-MRS value changes in moderate hyperbilirubinemia. Quantitative susceptibility mapping (QSM) is a relatively new MRI technology for quantifying the distribution of magnetic susceptibility in biological tissue (15). There are some factors that may affect the susceptibility. In adults, these changes are dominated by iron content and myelin, while in neonatal brains, other factors such as macromolecules and water-macromolecule exchange are also included (16, 17). Though the neonatal brain has little iron deposition and myelination occurs after birth, Zhong et al. detected susceptibility variation among brain regions (18). Zhang et al. (19) reported that deep gray nuclei in the neonate brain showed negative susceptibility. Moreover, some studies (9, 12) have shown that the neurotoxic effects of bilirubin may induce demyelination. Thus, we speculate that the susceptibility values of the nuclei of neonates with NHB would be decreased as areas in nuclei are susceptible to unconjugated bilirubin.

In this study, we explored the variability of susceptibility values and brain metabolites in patients with moderate NHB in comparison with controls and explored the correlations between susceptibility values, brain metabolites, and biochemical tests to ascertain whether moderate hyperbilirubinemia can induce neurological changes.

## 2 Materials and methods

### 2.1 Participants

Term infants with clinically diagnosed hyperbilirubinemia (4–11 days after birth) were recruited between June 2022 and February 2023. Term infants with neonatal hypoglycemia (4–10 days after birth, glucose level 1.9–2.6 mmol/L, and the hypoglycemia was corrected the second day after birth without any treatment) were recruited as the controls in this study and examinations were performed 5–7 days after correction of hypoglycemia. Written consent from the parents or legal guardians was obtained before all the tests. All MRI scans were obtained after infants naturally slept and without any medication. The time discrepancy between the blood sample results and the MRI scans was less than 2 days. The institutional review board approved this retrospective study. Infants with additional diagnoses (e.g., hypoxic ischemia, hemorrhage, or brain malformation), those with scans of poor image quality and abnormal image features (e.g., hyperintensity in basal ganglia), and those with typical clinical findings of kernicterus were excluded from this study.

### 2.2 Magnetic resonance imaging acquisition

MRI data were obtained using a Siemens 3.0T Magnetom Vida scanner. A 64-channel standard quadrature head coil was used. A 3D T1-weighted image was acquired using a magnetization-prepared rapid acquisition gradient-echo (MPRAGE) sequence on the sagittal plane with the following parameters: TR = 1,900 ms, TE = 2.98 ms, flip angle = 9, field of view = 256 × 256, and 1 mm^3^ isotropic resolution. The QSM sequences were acquired using the following: TR = 29 ms, TE = (5.36/10.19/15.02/19.85/24.68 ms), flip angle = 15, contrast = 5, field of view = 220 × 220, in-plane resolution = 0.7 × 0.7 mm^2^, slice thickness = 2.5 mm, and scan time = 4:50 min. Point resolved spectroscopy (PRESS) combined with the chemical shift imaging (CSI) sequences was used with the following parameters: TR = 1520 ms, TE = 135 ms, flip angle = 90°, averages = 3, volume of interest (VOI) dimension = 30 × 30 × 10 mm^3^ covering the region of the whole globus pallidus, bandwidth = 1,200 Hz, water suppression bandwidth = 50 Hz, and scan time = 6 min.

### 2.3 Biochemical tests

Blood samples were collected on the day of hospitalization in the NBE group and 5–7 days after glucose correction in the control group. Biochemical test results were collected, including total bilirubin (TBiL), indirect bilirubin (IBiL), direct bilirubin (DBiL), albumin (ALB), blood urea nitrogen (BUN), creatinine (CREA), uric acid (UA), alkaline phosphatase (ALP), free triiodothyronine (FT3), free thyroxine (FT4), and thyroid stimulating hormone (TSH).

### 2.4 QSM image preprocessing and regions of interest identification

QSM data were reconstructed using the MEDI Toolbox for field map estimation, background field removal with projection onto the diploe fields algorithm, and dipole inversion. Unwrapping was performed after field map estimation using Laplacian-based phase unwrapping. We registered the QSM maps to T1 images using FSL and then the T1 images were registered to ChildBrainAtlas (20, 21) and registered the QSM maps to the ChildBrainAltas and segmented them into 122 ROIs. QSM values for each ROI were extracted using data processing, analysis toolbox for brain imaging (DPABI). Since the basal ganglia are susceptible to unconjugated bilirubin in NBE, the ROI values in the basal ganglia region were included in the statistical processing. Thus, eight ROIs, including the left and right caudate nucleus (CN), the left and right globus pallidus (GP), the left and right putamen (PU), and the left and right thalamus were collected.

### 2.5 ^1^H MR spectroscopic preprocessing and data acquisition

^1^H MR spectroscopy data were obtained by using a linear combination model (LCmodel). Columns and rows were recognized automatically after raw data were loaded in the LCmodel and digital imaging and communications in medicine (DICOM) standards, and the voxels in the file starting at the top left were used to assess the globus pallidus region to quantify the metabolites. Glycerophosphocholine and phosphocholine (GPC and PCh, recorded as GPC*), creatine and phosphocreatine (Cr and PCr, recorded as Cr), glutamate and glutamine (recorded as Glx), and N-acetylaspartate (NAA) and N-acetylaspartylglutamate (NAAG) were indistinguishable, and combined signal intensities were used for further analysis (recorded as NAA*). Thus, the ^1^H metabolites with adequate reliability for quantification included GPC, myo-inositol (Ins), Cr, NAA, NAA*, and Glx with smaller than 20% of the estimation concentration and absolute data shift of less than 0.1 ppm. The meanings of the absolute concentrations of metabolites are unknown and the Cr concentration is relatively stable in most pathological brain conditions. Metabolites are expressed as a ratio relative to Cr.

### 2.6 Statistical analysis

The Mann–Whitney *U*-test was used to assess differences between the NHB and control groups in biochemical test results, QSM values, and ^1^H-MRS results. Differences in absolute concentrations of metabolism in ^1^H-MRS were also analyzed. Partial least square correlation (PLSC) analyses were performed to evaluate associations between biochemical test results and the susceptibility values and ^1^H-MRS results. A publicly available PLSC implementation in Matrix Laboratory (MATLAB) was used: https://github.com/danizoeller/myPLS. PLSC (22, 23) is a correlational technique in which analyses share information between two sets of data using the singular value decomposition (SVD) to determine the inertia (i.e., the sum of the singular values) of the covariance matrix of two original matrices (24). PLSC seeks the linear combinations of latent variables from two sets of data that maximally covariate with each other (25). Thus, the higher the value of singular value inertia observed, the greater the amount of shared information, and a stronger relationship between the two original matrices can be visualized. The PLSC analysis was computed as follows.

### 2.7 The correlation between biochemical tests and susceptibility values

PLSC was used to evaluate the association between the biochemical test results and susceptibility values in the NHB and control groups. A 25 × 11 matrix representing the biochemical test results was collected as X. Each row of X represents one participant, and each column is a biochemical test result. The susceptibility values in the basal ganglia region were also gathered into a 25 × 8 matrix as Y. The row in Y represents the participant, while the column is the susceptibility value. A cross-covariance matrix was calculated between X and Y. Singular value decomposition was then applied to this cross-covariance matrix, resulting in latent components. Each latent component was composed of a set of biochemical loadings and QSM loadings. Each loading indicated how strongly each biochemical result or susceptibility value contributed to the multivariate association of the biochemical results and magnetic susceptibility values. Furthermore, permutation and bootstrap tests were executed to determine the statistical significance and stability of the latent component. The permutation test was randomly repeated 1,000 times to obtain a null distribution of singular values. If the singular value of the sample is rare enough (*p* < 0.05), it is considered statistically significant. The stability of saliences was assessed by bootstrapping. Confidence intervals and bootstrap ratios can be derived from bootstrapping by repeating 500 times with replacement. If a zero value is not in the confidence interval of the salience of a variable, the variable is considered relevant. The bootstrap ratio was computed by dividing the mean distribution of the variable by its standard deviation. If the ratio was above 0.4 or below −0.4, then the latent component was considered robust. The same procedure was performed in the analysis of the biochemical results and susceptibility values in the control group.

### 2.8 The correlation between biochemical tests and metabolite ratios

PLSC was also applied to assess the correlation between biochemical tests and ^1^H-MRS results in the NHB and control groups. A 26 × 11 matrix representing biochemical test results was collected as X. Another 26 × 6 matrix of metabolite ratios was built as Y. Each row represented one participant and each column represented one metabolite ratio result. Matrix X was the same as that in the previous analysis. The computed procedure was similar to the previous procedure. Another PLSC analysis of the relationship between the biochemical tests and metabolite ratios in the control group was performed with the same procedure.

## 3 Results

### 3.1 Demographic and clinical characteristics

In total, 28 (12 girls and 16 boys) patients with NHB with moderate TSB levels (17.6–21.3 mg/mL) and 16 controls (9 girls and 7 boys) were enrolled. All the infants were born in term with full Apgar scores. No significant differences were found in age, birth weight, and gestational age between the two groups. Details are presented in Table 1. In the NHB group, three infants failed to obtain the QSM map while another two infants had uncompleted MRS scans. Thus, 25 infants with moderate TSB concentrations were enrolled in the susceptibility values analysis while the data of 26 infants with NHB were included in the following analysis of metabolite differences.

**Table 1:** Comparison of the demographic data.

Item	NHB group (*n* = 28)	Control group (*n* = 16)	*t*	*p*
Age (days)	5.39 ± 2.18	5.69 ± 1.30	0.491	0.626
Sex	12 girls, 16 boys	9 girls, 7 boys	–	–
Gestational age	39.03 ± 1.16	38.81 ± 0.85	0.373	0.711
Weight (g)	3,315.53 ± 331.61	3,360.31 ± 460.31	−0.672	0.505
Apgar score	10	10	–	–

### 3.2 Differences in biochemical test results, susceptibility values, and metabolite values between the NHB and control groups

The Mann–Whitney *U*-test showed that there were significant differences in TBiL, DBiL, IBiL, BUN, CREA, UA, and TSH between the two groups (*p* < 0.05). The susceptibility value of the left putamen was found to be significantly different between the NHB and control groups. The susceptibility values of the bilateral thalamus in the NHB group and the bilateral putamen and globus pallidus in the NHB and control groups showed negative values, which meant they were diamagnetic. The GPC absolute concentration in the NHB group was significantly decreased compared to that in the control group. No statistical differences were found in metabolite ratios between the two groups. Details are presented in Table 2.

**Table 2:** Comparison of the biochemical test results, susceptibility values, and metabolite values in the NHB group and control group.

Name	NHB group	Control group	*Z*	*p*
TBiL	315.95 (308.22, 348.25)	44.15 (39.45, 47.57)	5.465	**0**.**000**
DBiL	13.00 (11.50, 14.80)	8.15 (6.67, 8.97)	4.332	**0**.**000**
IBiL	304.65 (297.43, 333.30)	36.60 (33.58, 40.70)	5.465	**0**.**000**
ALB	36.05 (34.83, 37.38)	35.50 (34.32, 36.52)	0.842	0.400
BUN	2.16 (1.74, 2.89)	5.20 (3.37, 4.49)	−4.502	**0**.**000**
CREA	33.50 (27.00, 39.00)	58.50 (52.25, 64.75)	−5.274	**0**.**000**
UA	148.500 (119.00, 199.75)	344.00 (298.00, 400.25)	−5.088	**0**.**000**
ALP	183.00 (153.00, 203.00)	162.50 (141.50,190.75)	0.891	0.373
FT3	179.00 (150.5, 01.00)	162.50 (141.50, 190.75)	−0.427	0.669
FT4	5.74 (5.14, 6.13)	5.94 (5.07, 6.52)	1.695	0.097
TSH	23.73 (21.80, 26.26)	22.82 (20.87, 24.54)	4.026	**0**.**000**
THU_L	−1.65 (−6.37, 3.31)	0.02 (−3.04, 6.46)	−1.355	0.182
THU_R	−0.08 (−3.93, 3.76)	0.12 (−0.01,7.07)	−1.467	0.148
PU_L	−2.85 (−7.53, −1.53)	−0.90 (−3.21, 0.004)	−2.026	**0**.**043**
PU_R	−3.69 (−6.99, −0.48)	−3.62 (−7.51, −0.003)	−0.349	0.740
GP_L	−4.82 (−9.79, 1.44)	−0.97 (−5.51, −1.40)	−1.802	0.074
GP_R	−4.19 (−8.57, −0.19)	−3.14 (−8.17, −0.002)	−0.489	0.639
CN_L	0.08 (−2.62,3.28)	0.02 (−7.74, 2.28)	0.321	0.761
CN_R	0.43 (−3.57, 4.28)	0.62 (−0.01, 3.12)	−0.237	0.825
GPC (×10^−5^)	5.89 (5.43, 6.29)	6.50 (5.98, 7.25)	−2.465	**0**.**014**
Ins (×10^−4^)	2.54 (2.33, 2.95)	2.69 (2.38, 2.84)	−0.195	0.845
NAA (×10^−5^)	8.30 (7.57, 8.83)	8.83 (7.84, 9.04)	−1.098	0.272
GPC* (×10^−5^)	5.96 (5.67, 6.29)	6.50 (6.11, 7.25)	−2.501	**0**.**012**
Ins/Cr	3.31 (2.94, 3.53)	3.32 (2.77, 3.56)	0.130	0.896
NAA/Cr	1.07 (0.97, 1.12)	1.01 (0.97, 1.10)	0.806	0.420
NAA*/Cr	1.33 (1.22, 1.41)	1.28 (1.21, 1.39)	0.379	0.705
GPC/Cr	0.77 (0.73, 0.80)	0.79 (0.75, 0.86)	−7.712	0.087
Glx/Cr	1.54 (1.39, 1.65)	1.60 (1.44, 1.73)	−0.891	0.373

TBiL, total bilirubin; IBiL, indirect bilirubin; DBiL, direct bilirubin; ALB, albumin; BUN, blood urea nitrogen; CREA, creatinine; UA, uric acid; ALP, alkaline phosphatase; FT3, free triiodothyronine; FT4, free thyroxine; TSH, thyroid stimulating hormone; THU, thalamus; PU, putamen; GP, globus pallidus; CN, caudate nucleus; GPC, glycerophosphocholine; GPC*, glycerophosphocholine and phosphocholine; Cr, creatine and phosphocreatine; Ins, myo-inositol; NAA, N-acetylaspartate; NAA*, N-acetylaspartate and N-acetylaspartylglutamate; Glx, glutamate and glutamine. Significant differences (*p* < 0.05) are marked in bold.

### 3.3 Correlation between the biochemical test results and susceptibility values

**Figure 1:**
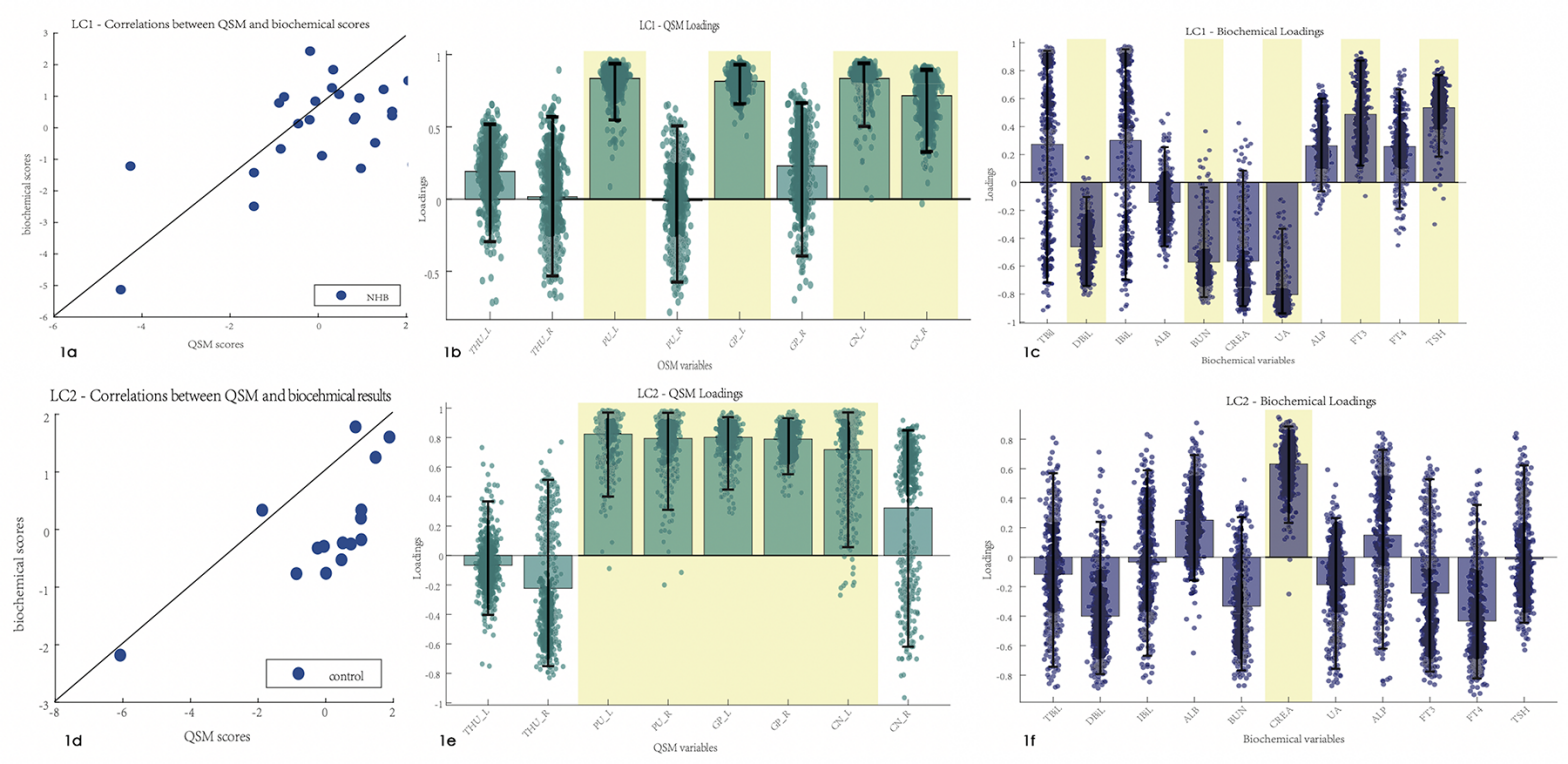
Correlations between the biochemical test results and susceptibility values. Error bars indicate the bootstrapped 5th to 95th percentiles. Robust results are indicated by a yellow background. **(a–c)** The scatter diagram, QSM loadings, and biochemical loadings of the NHB group. **(d–f)** The information of the control group. TBiL, total bilirubin; IBiL, indirect bilirubin; DBiL, direct bilirubin; ALB, albumin; BUN, blood urea nitrogen; CREA, creatinine; UA, uric acid; ALP, alkaline phosphatase; FT3, free triiodothyronine; FT4, free thyroxine; TSH, thyroid stimulating hormone; THU, thalamus; PU, putamen; GP, globus pallidus; CN, caudate nucleus.

PLSC analysis of the biochemical test results and susceptibility values identified one statistically significant latent component: latent component 1 revealed that increased susceptibility values of the left putamen, globus pallidus, and bilateral caudate nucleus were associated with decreased DBiL, BUN, and UA, and increased FT3 and TSH (*r* = 0.59 and *p* = 0.026, respectively) (Figure 1). PLSC analysis of the control group showed that increased susceptibility values of the bilateral putamen, globus pallidus, and left caudate nucleus were associated only with increased CREA in the biochemical test results.

### 3.4 Correlation between biochemical test results and metabolite ratios

**Figure 2:**
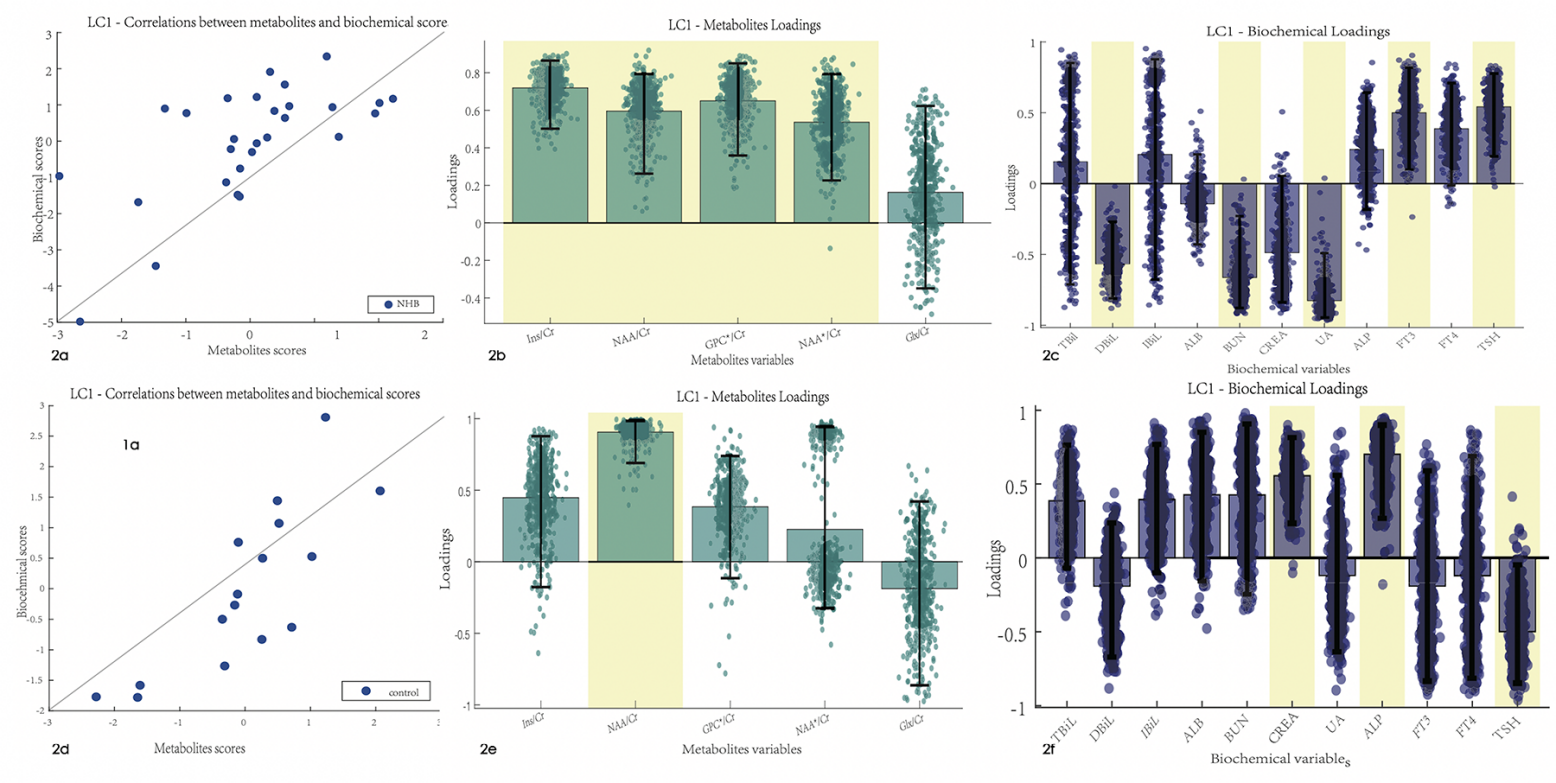
Correlation between the biochemical test and MRS results. Error bars indicate the bootstrapped 5th to 95th percentiles. Robust results are indicated by a yellow background. **(a–c)** The scatter diagram, QSM loadings, and biochemical loadings of the NHB group. **(d–f)** The information of the control group. TBiL, total bilirubin; IBiL, indirect bilirubin; DBiL, direct bilirubin; ALB, albumin; BUN, blood urea nitrogen; CREA, creatinine; UA, uric acid; ALP, alkaline phosphatase; FT3, free triiodothyronine; FT4, free thyroxine; TSH, thyroid stimulating hormone. Ins, myo-inositol; NAA, N-acetylaspartate; NAA*, N-acetylaspartate and N-acetylaspartylglutamate; Glx, glutamate and glutamine.

After PLSC analysis of the biochemical test results and metabolite ratios in the NHB group, latent component 1 was identified as statistically significant with *r* = 0.66 and *p*-value = 0.01. An association was found between the biochemical test results and metabolite ratios. Latent component 1 revealed that the Ins/Cr, NAA/Cr, and GPC/Cr ratios had a robust correlation with DBiL, BUN, UA, FT3, and TSH in the NHB group, with bootstrap CIs that did not cross the zero value (Figure 2). PLSC analysis of the control group indicated that latent component 1 was significant and revealed that the NAA ratio was correlated with CREA, ALP, and TSH in the biochemical test results.

## 4 Discussion

The present study aimed to explore whether moderate hyperbilirubinemia has an impact on brain metabolic function and susceptibility changes. It shows that the susceptibility value of the left putamen and the metabolite value (GPC absolute concentration) decreased in NHB group in comparison with the control group. Robust correlations between biochemical test results and susceptibility values and metabolite values were also found during the comparison. Decreased DBiL, BUN, and UA, and increased FT3 and TSH were found to be associated with increased susceptibility values and metabolite ratios in the NHB group in the PLSC analysis.

^1^H-MRS detects brain biochemistry changes *in vivo*. A previous study (13) reported that an elevation of Tau/Cr and Glx/Cr and a reduction of Cho/Cr in neonates with kernicterus were significantly different compared to published normal values. Lin et al. (11) also found increased Cho and GABA absolute concentrations in the case group compared to the normal control group. In our study, GPC concentrations in the NHB group were significantly decreased compared to those in the control group, which was in line with previous studies, while Tau and GABA were not found for the %SD > 20%, which was unacceptable reliability, and Glx/Cr had no significant difference. Wu et al. (26) found that the peak-area ratios of NAA/Cr and NAA/Cho in the basal ganglia were significantly lower in the NBE group than in the NHB and control groups, and they also found that the NAA/Cr ratio was not significantly different between the NHB and control groups. These findings may imply that a moderate elevation of TSB levels may have subtle effects on some brain biochemistry changes that can hardly be detected by ^1^H-MRS. Both Wu et al. and this study had a small sample of participants but the effects of a moderate elevation of TSB levels are still worth discussing. In this study, an association has been found between the metabolite ratios and biochemical results and reveals that the ratios of Ins, NAA, and GPC relative to Cr have a robust correlation with DBiL. To our knowledge, unconjugated bilirubin is responsible for neurotoxicity. Thus, we speculated that a correlation between metabolite ratios and unconjugated bilirubin should be found. However, after 1,000 permutation tests and 500 bootstrap tests, only DBiL was associated with these metabolite ratio changes. The reason for this is unknown and we did not exclude the fact that the limited number of participants may have contributed to this result.

The QSM has a unique high sensitivity for detecting subtle susceptibility changes in biological tissue (27). Susceptibility of deep gray nuclei is mainly influenced by iron content and myelination in adults, while in neonatal brains, these effects are dominated by myeline content (18). Few studies have discussed susceptibility values in infants in their first days of life with regard to their limited iron concentrations and myelination. Raab et al. (28) found that in the first 2 years after birth, susceptibility values increased rapidly, which indicates a combined effect of iron deposition and myelination. Ning et al. (29) found that the globus pallidus exhibited more rapid accumulations of iron but relatively slow accumulations in other nuclei by comparing the susceptibility values in deep gray matter nuclei from 1 month to 6 years. Otani et al. (30) found that susceptibility values in deep gray matter nuclei increased with age, and the normal development group showed higher susceptibility values than the delayed development group in the basal ganglia at an early postnatal age (<285 days). Deep gray nuclei, especially in the globus pallidus, are susceptible to free unconjugated bilirubin and may finally induce permanent gliosis. However, no previous studies have discussed the variations in brain magnetic susceptibility in NHB and the relationship between susceptibility values and biochemical test results. In this study, the susceptibility values of the putamen and globus pallidus revealed they were diamagnetic in both the NHB and control groups, which was in line with previous studies (18, 19). Moreover, the susceptibility value of the left putamen was significantly lower in the infants with NHB than in the control group. PLSC analysis showed that increased susceptibility values of the putamen, globus pallidus, caudate nucleus, and thalamus had a moderate correlation with decreased DBiL and increased TSH concentrations in the NHB group. In contrast, a completely different trajectory was found in the control group. Thus, we concluded that moderate hyperbilirubinemia may have compound effects on susceptibility changes in deep gray nuclei.

There are some limitations in this study. First, a limited number of participants was used due to the rare clinical conditions. This has an influence on the reproducibility and generalizability of the results. Thus, a larger number of participants should be enrolled in future studies. Second, infants with hypoglycemia were utilized as the control group but examinations were conducted after blood glucose was corrected, which could potentially induce some errors in the results. Third, regions involved in neurotoxicity in NHB include the globus pallidus, substantia nigra reticulata, subthalamic nuclei, vestibular and oculomotor nuclei, hippocampus, and cerebellum. In this study, only the values of the basal ganglia areas were calculated. Fourth, the TSB levels in the NHB group varied and no subtypes of NHB were discussed in this study which may have influenced the results. Further study of the subtypes of NHB should be explored in the future. Moreover, only 1 month of follow-up was conducted during this examination, and the assessment consisted of biochemical tests and physical examinations. Thus, whether these moderate hyperbilirubinemia-induced metabolic changes had an impact on the infants' development is unknown. A long-term follow-up should be arranged for a complete evaluation of sequelae.

## 5 Conclusion

This study demonstrated that moderate hyperbilirubinemia can induce metabolic and susceptibility changes in an infant’s brain (e.g., decreased susceptibility values and metabolite values) and these metabolic changes have a correlation with biochemical test results.

## Data Availability

The original contributions presented in the study are included in the article/Supplementary Material, further inquiries can be directed to the corresponding author/s.
